# Reducing the Impact of the Next Influenza Pandemic Using Household-Based Public Health Interventions

**DOI:** 10.1371/journal.pmed.0030361

**Published:** 2006-08-08

**Authors:** Joseph T Wu, Steven Riley, Christophe Fraser, Gabriel M Leung

**Affiliations:** 1 Department of Community Medicine and School of Public Health, Faculty of Medicine, University of Hong Kong, Hong Kong, Special Administrative Region, People's Republic of China; 2 School of Industrial and Systems Engineering, Georgia Institute of Technology, Atlanta, Georgia, United States of America; 3 Department of Infectious Disease Epidemiology, Imperial College London, London, United Kingdom; National Institute for Public Health and the Environment, Netherlands

## Abstract

**Background:**

The outbreak of highly pathogenic H5N1 influenza in domestic poultry and wild birds has caused global concern over the possible evolution of a novel human strain [1]. If such a strain emerges, and is not controlled at source [2,3], a pandemic is likely to result. Health policy in most countries will then be focused on reducing morbidity and mortality.

**Methods and Findings:**

We estimate the expected reduction in primary attack rates for different household-based interventions using a mathematical model of influenza transmission within and between households. We show that, for lower transmissibility strains [2,4], the combination of household-based quarantine, isolation of cases outside the household, and targeted prophylactic use of anti-virals will be highly effective and likely feasible across a range of plausible transmission scenarios. For example, for a basic reproductive number (the average number of people infected by a typically infectious individual in an otherwise susceptible population) of 1.8, assuming only 50% compliance, this combination could reduce the infection (symptomatic) attack rate from 74% (49%) to 40% (27%), requiring peak quarantine and isolation levels of 6.2% and 0.8% of the population, respectively, and an overall anti-viral stockpile of 3.9 doses per member of the population. Although contact tracing may be additionally effective, the resources required make it impractical in most scenarios.

**Conclusions:**

National influenza pandemic preparedness plans currently focus on reducing the impact associated with a constant attack rate, rather than on reducing transmission. Our findings suggest that the additional benefits and resource requirements of household-based interventions in reducing average levels of transmission should also be considered, even when expected levels of compliance are only moderate.

## Abstract 2

Voluntary household-based quarantine and external isolation are likely to be effective in limiting the morbidity and mortality of an influenza pandemic, even if such a pandemic cannot be entirely prevented, and even if compliance with these interventions is moderate.

## Editors' Summary

**Background.**

Naturally occurring variation in the influenza virus can lead both to localized annual epidemics and to less frequent global pandemics of catastrophic proportions. The most destructive of the three influenza pandemics of the 20th century, the so-called Spanish flu of 1918–1919, is estimated to have caused 20 million deaths. As evidenced by ongoing tracking efforts and news media coverage of H5N1 avian influenza, contemporary approaches to monitoring and communications can be expected to alert health officials and the general public of the emergence of new, potentially pandemic strains before they spread globally.

**Why Was This Study Done?**

In order to act most effectively on advance notice of an approaching influenza pandemic, public health workers need to know which available interventions are likely to be most effective. This study was done to estimate the effectiveness of specific preventive measures that communities might implement to reduce the impact of pandemic flu. In particular, the study evaluates methods to reduce person-to-person transmission of influenza, in the likely scenario that complete control cannot be achieved by mass vaccination and anti-viral treatment alone.

**What Did the Researchers Do and Find?**

The researchers developed a mathematical model—essentially a computer simulation—to simulate the course of pandemic influenza in a hypothetical population at risk for infection at home, through external peer networks such as schools and workplaces, and through general community transmission. Parameters such as the distribution of household sizes, the rate at which individuals develop symptoms from nonpandemic viruses, and the risk of infection within households were derived from demographic and epidemiologic data from Hong Kong, as well as empirical studies of influenza transmission. A model based on these parameters was then used to calculate the effects of interventions including voluntary household quarantine, voluntary individual isolation in a facility outside the home, and contact tracing (that is, asking infectious individuals to identify people whom they may have infected and then warning those people) on the spread of pandemic influenza through the population. The model also took into account the anti-viral treatment of exposed, asymptomatic household members and of individuals in isolation, and assumed that all intervention strategies were put into place before the arrival of individuals infected with the pandemic virus.

  Using this model, the authors predicted that even if only half of the population were to comply with public health interventions, the proportion infected during the first year of an influenza pandemic could be substantially reduced by a combination of household-based quarantine, isolation of actively infected individuals in a location outside the household, and targeted prophylactic treatment of exposed individuals with anti-viral drugs. Based on an influenza-associated mortality rate of 0.5% (as has been estimated for New York City in the 1918–1919 pandemic), the magnitude of the predicted benefit of these interventions is a reduction from 49% to 27% in the proportion of the population who become ill in the first year of the pandemic, which would correspond to 16,000 fewer deaths in a city the size of Hong Kong (6.8 million people). In the model, anti-viral treatment appeared to be about as effective as isolation when each was used in combination with household quarantine, but would require stockpiling 3.9 doses of anti-viral for each member of the population. Contact tracing was predicted to provide a modest additional benefit over quarantine and isolation, but also to increase considerably the proportion of the population in quarantine.

**What Do These Findings Mean?**

This study predicts that voluntary household-based quarantine and external isolation can be effective in limiting the morbidity and mortality of an influenza pandemic, even if such a pandemic cannot be entirely prevented, and even if compliance with these interventions is far from uniform. These simulations can therefore inform preparedness plans in the absence of data from actual intervention trials, which would be impossible outside (and impractical within) the context of an actual pandemic. Like all mathematical models, however, the one presented in this study relies on a number of assumptions regarding the characteristics and circumstances of the situation that it is intended to represent. For example, the authors found that the efficacy of policies to reduce the rate of infection vary according to the ease with which a given virus spreads from person to person. Because this parameter (known as the basic reproductive ratio, *R*_0_) cannot be reliably predicted for a new viral strain based on past epidemics, the authors note that in an actual influenza pandemic rapid determinations of *R*_0_ in areas already involved would be necessary to finalize public health responses in threatened areas. Further, the implementation of the interventions that appear beneficial in this model would require devoting attention and resources to practical considerations, such as how to staff isolation centers and provide food and water to those in household quarantine. However accurate the scientific data and predictive models may be, their effectiveness can only be realized through well-coordinated local, as well as international, efforts.

**Additional Information.**

Please access these Web sites via the online version of this summary at http://dx.doi.org/10.1371/journal.pmed.0030361.

• World Health Organization influenza pandemic preparedness page

• US Department of Health and Human Services avian and pandemic flu information site

• Pandemic influenza page from the Public Health Agency of Canada

• Emergency planning page on pandemic flu from the England Department of Health

• Wikipedia entry on pandemic influenza with links to individual country resources (note: Wikipedia is a free Internet encyclopedia that anyone can edit)

## Introduction

The basic reproductive number *R*_0_ is the average number of people infected by a typically infectious individual in an otherwise susceptible population [5]. If the basic reproductive number is greater than one, the disease has the potential to spread. If it is less than one, the disease will die out after only a few generations. The next influenza pandemic will start when a novel strain of influenza evolves with *R*_0_ > 1 in humans. Wherever in the world the novel strain evolves, with modern news services and electronic communication, there will be a period of time during which the disease is not present in some large populations but is known to be spreading in other more remote locations. This presents a window of opportunity for implementing interventions to reduce *R*_0_ prior to the introduction of the pandemic strain from those remote populations. Although *R*_0_ is sometimes considered to be intrinsic to a pathogen, it is also dependent on the behavior of the host population and can vary across time. For instance, for the severe acute respiratory syndrome that re-emerged during the winter of 2003–2004 [6], since there was no significant sustained transmission, *R*_0_ was likely to have been lower than when the virus first circulated during the previous year [7]. The first-wave infection attack rate (IAR) is defined to be the proportion of the population infected during the initial epidemic (i.e., first year) of the circulation of a novel pathogen and includes both symptomatic and asymptomatic infection. Specifically, the IAR for pandemic influenza does not include infections arising during subsequent years. Public health policy in disease-free populations prior to the arrival of pandemic influenza should aim to reduce *R*_0_, thus reducing the IAR. The IAR will not be known until after the first wave has passed and will be either measured directly, using serological surveys, or estimated from case reports and knowledge of the proportion of infections that were symptomatic.

Let 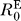 be the effective basic reproductive number for pandemic influenza in a host population undergoing some preventive intervention prior to its arrival. Recent analysis of clinical trial data [8] suggests that 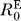 = *R*_0_/3.6 can be achieved with prolonged mass prophylaxis with oseltamivir [2]. Therefore, pandemic strains with *R*_0_ < 3.6 could be controlled, but this would require a drug stockpile of 56 doses per member of the population for only 8 wk of protection, which would likely not be long enough and is currently unfeasible. However, even in circumstances when an outbreak is not controlled, the impact of public health interventions that reduce 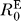 would still be substantial. Using a very simple model of disease spread, a precise relationship between IAR and 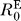 can be defined (see Figure S3). For high values of 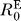 , IAR is close to one, but for values of 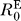 closer to one, IAR is much lower. Any policies that significantly reduce infectivity and/or susceptibility, over and above what would be the natural reaction of the population, will have a similar effect. Therefore, the potential benefits are large from apparently small reductions in low values of *R*_0_, even when complete control is not achieved.

**Figure 1:**
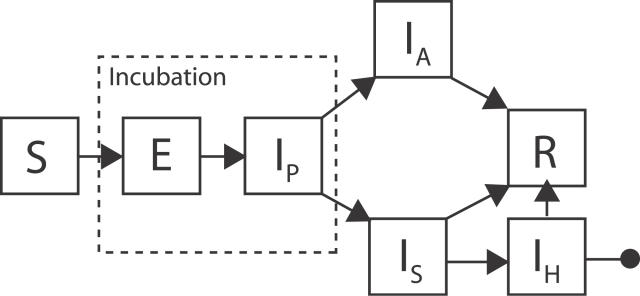
The Natural History Assumed for Pandemic Influenza Individuals progress from S (susceptible) through E (exposed but not yet infectious), I_P_ (infectious but not yet symptomatic), I_A_ (infectious and asymptomatic), I_S_ (infectious and symptomatic), I_H_ (infectious and suffering symptoms severe enough to be hospitalized), and R (recovered and presumed immune). All deaths occur in the I_H_ stage. We used recent results [2] derived from a symptom-based household study [15] for the waiting time of the combined E and I_P_ stages: it is distributed according to an offset Weibull with offset +0.5 d, mean 1.48 d (including the offset), and standard deviation 0.47 d. The duration of the I_P_ stage was assumed to be fixed at 0.5 d. The duration of the I_S_ stage was set to be 5/3 that of the I_A_ stage, but the absolute duration of both stages was determined by the generation time, *T*_g_ (see main text and Table 1). In the model, 33% of infections were never symptomatic, which is consistent with sources: a basic reproductive number of 1.8 [2] with a 50% case attack rate [24], and observations from deliberate infections of humans with H1N1 [25]. We assumed that 6.0% of symptomatic infections resulted in hospitalization and 17.2% of these resulted in death (nonpandemic data for community-acquired pneumonia extracted from the Hospital Authority Integrated Patient Administrative System, Hospital Authority, Hong Kong Special Administrative Region, 2002). The 6% hospitalization rate is consistent with the overall 1918 pandemic mortality rate (derived from analyses of the 1918 pandemic; see Discussion)—67% of infections being symptomatic—and with the case fatality rate for all community-acquired pneumonia admissions to Hong Kong public hospitals. We assumed that all children and 50% of adults stay at home when symptomatic with influenza—even when no interventions are in force (consistent with [20]). This assumption is to ensure that the impact of quarantine is not overestimated. Note that this parameter was not included in the sensitivity analyses as its impact was dominated by θ, the proportion of presymptomatic or asymptomatic transmission. Half of transmission outside the home was assumed to be in the peer group, the other half was between random pairs of infector and infectee who would not have been able to name each other during a contact tracing interview.

Here, we estimate the expected reduction in IAR for different household-based interventions using a mathematical model of influenza transmission within and between households. Household-based interventions, such as voluntary quarantine, isolation, and the provision of prophylactic anti-virals, may be able to reduce the IAR substantially without consuming resources at the same rate as nontargeted population-level interventions. Here, “quarantine” refers to the segregation, within their own homes, of household contacts of a suspected case from other members of the community. We use the term “isolation” for when compliant symptomatic individuals are removed from their household to a separate facility. We also consider contact tracing, where individuals are asked to name people who they may have infected, and those individuals are notified and asked to take precautionary measures. In order to estimate the impact of household-based interventions on IAR, a more detailed representation of the population structure is required. Therefore, we use an individual-based stochastic model of influenza transmission (Figure 1) with explicit household, peer-group, and community settings. Although substantial progress has been made in recent years [9–12], we were unable to derive useful analytical approximations describing the behavior of the required infection and intervention processes.

**Table 1:**
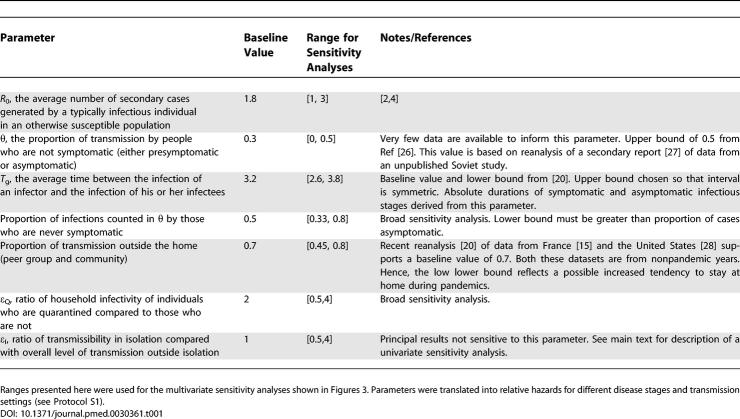
Assumptions for Key Unknown Transmission Parameters

## Methods

The distribution of household sizes and the average numbers of children in households of different sizes were simulated to be consistent with Hong Kong [13]. We made all interventions active prior to the arrival of the infected individuals, and we challenged the system with a constant introduction of 1.5 infected individuals per day per 100,000 people for 365 d. Our results showed no significant sensitivity when the importation rate was proportional to an epidemic curve (see Figure S1). Susceptible individuals reported with influenza-like illness, caused by something other than the pandemic influenza strain, at a constant rate of 74 per day per 100,000 people. This provided a constant stream of false positives, which ensures that the number of households in quarantine in the early stages of the epidemic is not underestimated. However, it should be noted that the relative benefits derived from testing are higher for higher rates of false positives. The rate we used is approximately equal to the peak reporting rate of influenza-like illness in the Hong Kong primary care setting during 2004 (Hong Kong Centre for Health Protection; http://www.chp.gov.hk/sentinel.asp?lang=en&id=292&pid=44&ppid=26). These nonpandemic cases were symptomatic for 3 d on average.

The hazard of infection from an infectious person to a susceptible person in a household was set to be inversely proportional to household size, reflecting recent findings for endemic influenza household transmission dynamics [14]. Model-generated household attack rates were consistent with recent empirical studies [15], given uncertainties in the degree of community transmission present in those studies (see Figure S2). Workplaces and schools were represented as large, highly connected peer groups. A further substantial proportion of transmission, termed “community” transmission, was assumed to be outside of the peer group and the home. Large network neighborhood sizes and substantial community transmission are conservative assumptions with respect to the efficacy of contact tracing: they penalize contact tracing without significantly affecting other interventions. A formal definition of the transmission model is given in Protocol S1. As there was no spatial component in this model, our results will overestimate the speed of the epidemic in geographically dispersed populations. However, large countries will suffer importation of infectious individuals in all regions, and pandemic strains will spread rapidly between large cities. Therefore, it is unlikely that geographical heterogeneities will last longer than 1 or 2 wk in large countries such as the United States [16], which suggests that there will be little opportunity for the use of spatially heterogeneous intervention strategies.

An integrated process of voluntary household quarantine, voluntary individual isolation, anti-viral administration, and contact tracing was used to predict the impact of household-based intervention policies. If an individual complied with household quarantine, their infectiousness to other household members changed by a factor of ɛ_Q_. Because quarantine increases the average time spent at home substantially for most people, the value of this parameter may be greater than unity (ɛ_Q_ = 2 at baseline; see Table 1). Also, the level of transmission in isolation may be higher than elsewhere. We assumed that the degree of transmission in isolation was a factor of ɛ_I_ greater (ɛ_I_ = 1 at baseline; see Table 1), i.e., the basic reproductive number inside transmission was equal to ɛ_I_ *R*_0_ (see Protocol S1). Note that we assumed that for all policies, those individuals with symptoms severe enough to be hospitalized (see Figure 1) would be isolated. Hence, policies without explicit isolation elements used isolation resources. Also, we assumed that all those in isolation received anti-viral treatment. Hence, policies without explicit anti-viral elements used anti-viral doses. We modeled compliance at the individual level: a symptomatic individual in a household that was not quarantined decided for herself if she reported, but the other members of her household made independent decisions for themselves. We defined *p*_c_ to be the probability of compliance.

These interventions were implemented using the following algorithm.

Step 1: an individual from households not in voluntary quarantine had the opportunity to enter the program via one of the following three routes: she developed symptoms, she was contacted through contact tracing, or she was hospitalized. We assumed she volunteered and actually reported with probability *p*_c_ for symptoms and contact tracing, and with probability one for hospitalization. She complied with the program until released. After release, individuals were not bound by previous decisions to join or not join, i.e., they could choose again.

Step 2: each other member of her household complied with intervention instructions with probability *p*_c_.

Step 3: after a delay of 1 d, all compliant nonsymptomatic household members took one dose of prophylactic anti-virals per day when anti-viral policies were in effect. Symptomatic household members took two doses of anti-virals per day.

Step 4: if contact tracing was in effect, each compliant adult member of the household named, on average, five members of their peer group, if she had not been asked to name contacts before.

Step 5: if isolation was in effect, newly found symptomatic individuals who were compliant voluntarily entered isolation with probability *p*_c_ after a delay of 1 d. If an isolated individual no longer showed symptoms after 3 d, she was released from isolation and joined her household, which might be quarantined. Otherwise, she was isolated for a further 3 d. This cycle repeated until she no longer showed symptoms or died (see Figure S4 for the distribution of durations of quarantine for different policies).

Step 6: isolated individuals were given two doses of anti-virals per day, without a delay, in all simulations, regardless of the policy for the use of anti-virals in households.

Step 7: if contact tracing was in effect, contacts (if known and not already in the program) of all newly found symptomatic or hospitalized household members were traced with a mean delay of 1 d.

Step 8: if there had been no new symptoms in compliant household members or hospitalizations of any household members for 7 d, the quarantined household was released from the program at the end of that period. Otherwise, we returned to Step 5 at the time new symptoms or hospitalizations occurred.

## Results

### Baseline Scenarios

**Figure 2:**
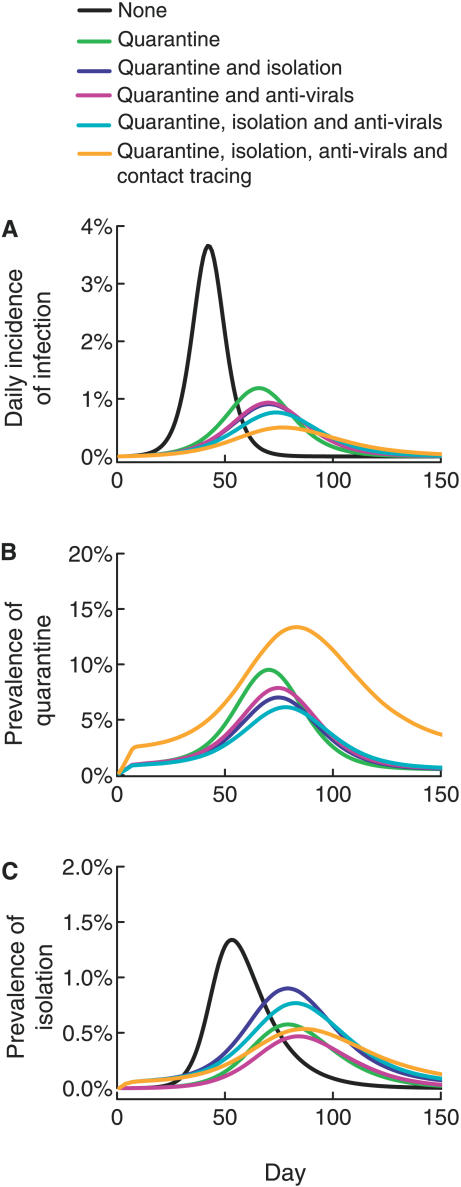
Baseline Transmission (None) and Five Intervention Scenarios: Q, QI, QA, QIA, and QIAC (A) shows the incidence of infection, (B) the percentage of the population living in homes that were quarantined, and (C) the percentage of the population in isolation. We assumed that infectivity within households increased by 100% for each individual who complied with quarantine. For peer groups, quarantine reduced both transmissibility and susceptibility to 25% of the nonquarantined level. Therefore, the rate of transmission decreased by 75% within a peer group when one of two infected individuals complied with quarantine and by 96% if both did. For the results presented here, we assumed a compliance rate of 50% (see Methods). If individuals complied, they were not infectious to the wider community. When anti-viral treatments were used they reduced susceptibility to 30% of its baseline value and transmissibility to 69% of its baseline value. These are the conservative bounds of the 95% confidence intervals from analyses of clinical trial data for oseltamivir [8]. Results presented here are averages of 100 realizations in a population of 1,000,000 individuals, but there was no significant change for populations of 500,000 or 2,000,000. The 95% stochastic prediction intervals (not shown) do vary with population size, but are narrow (less than 5% deviation from mean values) for simulations in populations of 1,000,000.

Model simulations show that with compliance rates of 50% all intervention policies (Figure 2) would have an impact on the baseline transmission scenario (Table 1). Although none were capable of complete control, all policies substantially reduced IAR. The baseline IAR of 74% was reduced to 49% when voluntary household quarantine (Q) alone was in effect. While voluntary quarantine with moderate compliance was not universally effective for all household sizes and transmission settings, it was highly effective in preventing transmission into the community from larger households, especially those with symptomatic index cases (see Table S1). However, the peak proportion of the population living in homes that were quarantined, even with only 50% program compliance, was 9.6%. The addition of voluntary individual isolation to Q (QI) further reduced the IAR to 43%, and the peak number of individuals living in homes that were quarantined decreased to 7.1%. Moreover, this policy provided an incentive for households to participate: presumed-infectious individuals could expect to be prioritized for health care by entering isolation and would protect household members by leaving the home. However, this approach required isolation facilities for up to 0.9% of the population at the peak of the epidemic.

We considered the use of anti-virals with Q (QA) as an alternative to isolation. This policy had a similar efficacy to QI (IAR 44%) at a cost of 3.9 doses of anti-viral per member of the population but, as would be expected, with a much smaller peak level of isolation of 0.5%. The use of anti-virals in addition to QI (QIA) further reduced the IAR to 40% and the peak proportion of the population living in homes that were quarantined to 6.2%. Finally, the addition of contact tracing to QIA (QIAC) reduced the IAR to 34% but increased the proportion of the population in quarantine considerably compared with all other policies. The additional requirements of contact tracing are unlikely to be justified other than when the reproductive number is reduced to near one by other interventions. The prevalence of quarantine and isolation (Figure 2B and 2C) determines the resources required by these programs over time, e.g., the total prevalence of quarantine and isolation on a given day determines the number of anti-viral doses that would need to be distributed when QIA was in effect. Note that the overall efficacy of interventions is reduced if interventions are delayed until 5% of the population have experienced symptomatic infection. However, the impact is minimal if the results are adjusted for the effective population size of 92.5% (see Figure S6).

### Sensitivity Analyses

**Figure 3:**
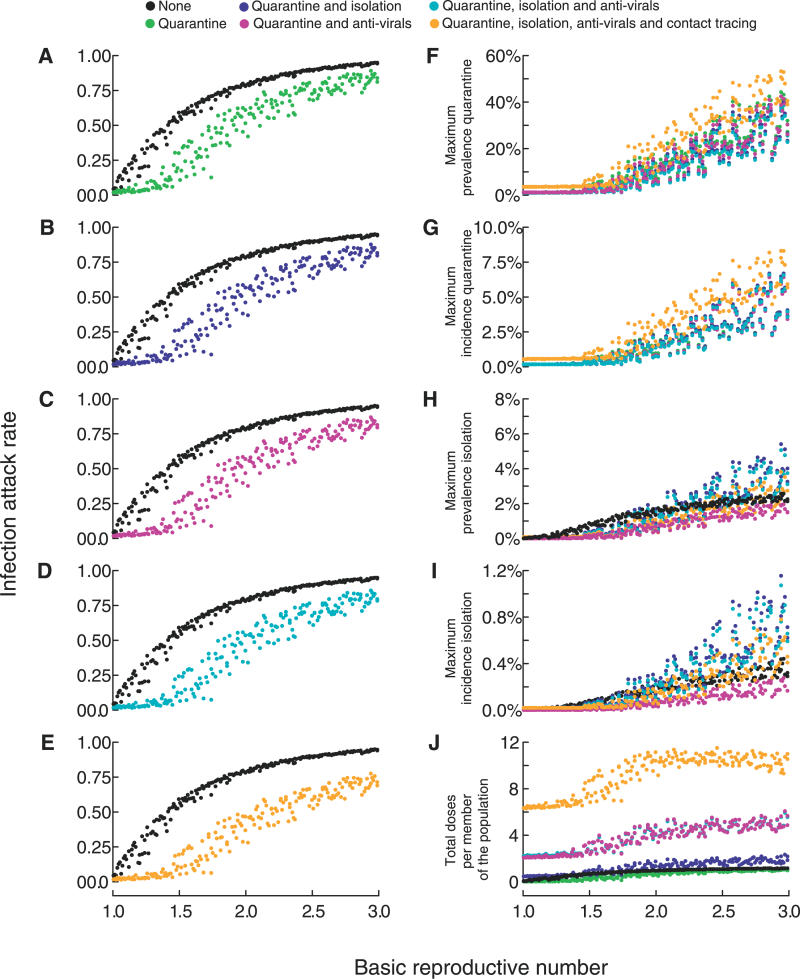
Multivariate Sensitivity Analyses (A–E) The efficacy of different household-based intervention policies (same color key as in Figure 2) compared to taking no action for 200 random samples, selected using a Latin hypercube [17], across a multivariate range of transmission parameters (see Table 1). The *y*-axes show the expected IAR for the first wave. Variation in IAR for a given *R*_0_ is driven by variation in other transmission parameters rather than by stochastic variation. (F–J) The maximum prevalence and incidence of quarantine and isolation and the total number of drug doses used per member of the population (same color key as in [A–E]). For maximum values, the average of individual realization maximums is used as the overall maximum. Incidences are daily. We assume that all isolated individuals (including those who are hospitalized) receive anti-viral treatment. Results presented here are averages of 100 realizations in a population of 500,000 individuals. Sub-samples at 1,000,000 show no significant differences in these patterns. *R*_0_ values are based on 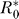 , the average number of secondary cases generated by an individual chosen at random in an otherwise susceptible population: 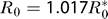 (see Figure S3).

As the influenza strain that will cause the next pandemic has not yet been observed, it is not possible to estimate its level of transmissibility (other than by using historical data from other strains [2,4]) or the balance of transmission between different settings. A multivariate range of plausible transmission scenarios (Table 1) represents both scientific uncertainty about the nature of influenza transmission across different populations and genuine stochasticity associated with a single evolutionary event. Even the most meticulous preparedness plan should be robust against a range of transmission scenarios. We used extensive Latin hypercube sampling [17] to conduct sensitivity analyses, the results of which suggest that variation in the efficacy of policies in reducing the attack rate will be dominated by the basic reproductive number, *R*_0_ (Figures 3A–3D and S3). Further, all interventions were considerably more effective and used fewer resources for lower values of *R*_0_. Although current low estimates of *R*_0_ [2,4] (based on historical data) are encouraging, in the event of a pandemic, rapid estimates of *R*_0_ based on contemporaneous data from early national epidemics will be crucial for finalizing public health responses elsewhere.

The results presented in Figure 3 suggest that the efficacy of QA was not substantially less than that of QIA for most parameter combinations. It is interesting that the potential for increased transmission in isolation did not seem to decrease substantially the efficacy of the QI strategy (ɛ_I_, the ratio of transmissibility in isolation to overall transmissibility outside isolation, varied from 0.5 to 4). In order to explore this finding further, we performed a univariate sensitivity analysis of the impact of changes in the relative transmissibility in isolation, ɛ_I_ (baseline parameter values: ɛ_I_ = 1, IAR 43%), on the efficacy of the QI policy. We found that even with isolation transmissibility levels ten times greater than those outside isolation, the QI policy was still effective (ɛ_I_ = 10, IAR 45%) when compared with Q alone (IAR 49%). This result occurred because the overall proportion of susceptible individuals entering isolation was low. Note that this proportion may have been high during the initial stages (as perhaps occurred in some settings during the 2003 SARS outbreak) but would likely be small when averaged over the entire course of the epidemic.

**Figure 4:**
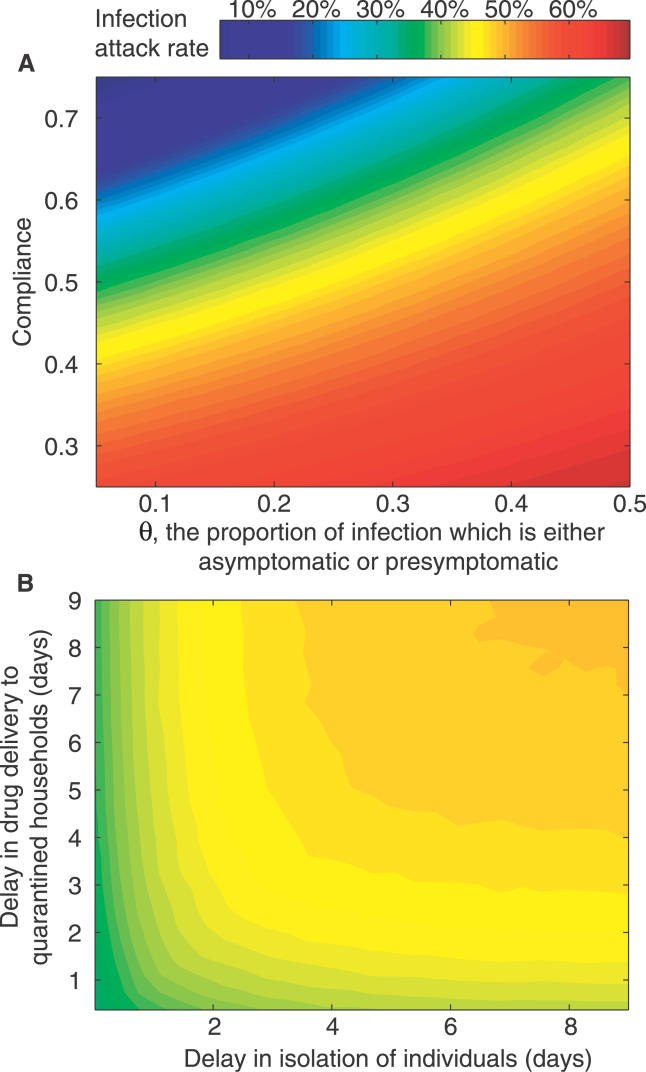
Bivariate Sensitivity Analyses (A) The sensitivity of our estimates of the efficacy of Q to changes in the compliance rate and to changes in θ, the proportion of infections that are either asymptomatic or presymptomatic. (B) The sensitivity of our estimates of the efficacy of QIA to changes in the delay in the delivery of drugs to quarantined households and to changes in the delay in the isolation of individuals. All other parameters are held constant at their baseline values (see Figure 1 and Table 1).

All estimated reductions in IAR were sensitive to the population compliance rate, *p*_c_, and to θ, the proportion of transmission that was either asymptomatic or presymptomatic (Figure 4A). Values of *p*_c_ = 50% and θ = 30% were assumed for baseline intervention scenarios (Table 1; Figures 2 and 3). Our estimated changes in IAR were also sensitive to the delay in the provision of anti-virals to households and to the delay in the isolation of symptomatic individuals (Figure 4B), although less so than to *p*_c_ and θ. In deciding whether to implement any or all of the policies described here, local public health officials may wish to consider available epidemiological data (to assess *R*_0_ and θ) and also estimate the levels of compliance that could be achieved for the different options in their populations. As compliance may be higher for policies that provide immediate benefits to the individual, we suggest that compliance will be low for Q, higher for QI and QA, and higher still for QIA. It is likely that the provision of anti-viral prophylaxis and treatment would increase compliance substantially. Our baseline assumption of 50% compliance is intended to be conservative. Intuitively, it seems likely that household-based interventions would work with high levels of compliance. Here, we have shown that even moderate levels of compliance would allow household-based public health interventions to be effective. Also, the marginal benefits of the use of anti-virals or isolation (policies QI, QA, and QIA versus Q) may not be justified if the average times for the provision of these services exceed 3 to 4 d, given that the quarantine period is set at 7 d.

Levels of compliance with quarantine and isolation would likely be improved in the early and late stages of the epidemic by the availability of a viable diagnostic. During the middle stage of the epidemic, the incidence of pandemic influenza will be much higher than that of other “background” respiratory infections. During this period, influenza-like symptoms will be an accurate indicator of infection with the pandemic strain. We also considered the impact of virological testing as a diagnostic support for these policies (see Protocol S1). However, current low throughput (limited by both laboratory infrastructure and supplies of reagent) and low test sensitivity (due mainly to difficulties in obtaining adequate specimens outside of specialized care settings) meant that it was not a worthwhile addition. If an inexpensive, easy-to-perform, rapid, and accurate test were to become available, it would have a significant impact on transmission and on peak levels of quarantine when used as part of a wider household-based program (Figure S5).

## Discussion

We have shown that, for lower transmissibility strains of pandemic influenza, the combination of household-based quarantine, isolation of cases outside the household, and targeted prophylactic use of anti-virals will be highly effective and likely feasible across a range of plausible transmission scenarios, even with only moderate levels of compliance. Further, we have quantified the resources consumed by this and similar policies in terms of numbers of people quarantined, numbers of people isolated, and doses of anti-virals required.

Sequence and phylogenetic analyses of the complete genome of the 1918 human influenza strain suggest it was closely related to avian strains [18]. In addition, recent analyses of the first wave of that pandemic to pass through New York City have estimated an overall excess mortality within the general population of 0.5% [19]. With an IAR of 74% (consistent with a basic reproductive number of 1.8), this corresponds to an infection mortality rate of 0.7%. Therefore, if we assume that the natural history of the next pandemic strain will be similar to that of the 1918 strain, a reduction in IAR from 74% to 40% would avert 16,000 deaths during the period of the initial wave of the pandemic in a city the size of Hong Kong (6.8 million people). Our results suggest that such a reduction could be achieved using the combination of voluntary quarantine, individual isolation, and anti-viral therapy. The use of isolation on such a large scale may be somewhat controversial, given the infrastructure requirements of such a policy. Therefore, for populations with large stockpiles of anti-virals available, the marginal benefit of the additional use of isolation may not be justified. However, there will be populations for which the large-scale stockpiling of anti-virals is not feasible and for which individual isolation represents the best possible addition to household quarantine.

Our results build on previous modeling studies of pandemic influenza that focused primarily on the possibility of containment using geographically targeted anti-viral therapy [2,3]. Here, and in two other recent studies [20,21], effective strategies have been identified for mitigation rather than containment. The key outcome of mitigation studies is the reduction in IAR, rather than the likelihood of complete control. Given that many epidemiological parameters associated with the next influenza pandemic are unknown, comparison of results from different modeling studies is not straightforward. Ferguson et al. [20] assumed that only infections that were severe enough to require medical care (50%) would be reported and could be used to trigger interventions. We assumed that during the next pandemic, 67% (all symptomatic) of infections could be reported and used to trigger interventions. Our more optimistic assumption about the symptomatic rate was balanced by assuming that only 50% of symptomatic cases would comply with our policies and report, whereas Ferguson et al. [20] assumed that 90% of clinical cases would receive anti-viral treatment (with their household receiving anti-viral prophylaxis) and that 50% of households would comply with quarantine. If we had assumed a 50% clinical attack rate rather than 67%, we would have estimated a reduction in symptomatic attack rate from 37% to 22% for the policy of QA. This estimate is consistent with the reduction in attack rate from 34% to 20% in Figure 3 of Ferguson et al. [20]. However, our results are not consistent with Table 2 of Germann et al. [21], in which a 10-fold reduction in the numbers of ill people is reported for the use of targeted anti-viral prophylaxis (60% case ascertainment, *R*_0_ = 1.9, unlimited supply of anti-virals). This large discrepancy is most likely due to the optimistic nature of their policy: they assume that households, household clusters, schools, and workplaces can be targeted very efficiently for prophylactic anti-viral therapy. We suggest that a highly efficient contact tracing process would be required to achieve high levels of coverage between socially connected households in modern urban populations, and, further, our results suggest that such a process would require unfeasibly large numbers of households to be recruited during short periods of time.

Individual decisions to comply with available quarantine or isolation programs, or to choose between them, will be substantially influenced by household structure, perceived benefits, and the presumed infection status of household members. For example, young children are very unlikely to enter isolation alone at any stage during the outbreak, whereas a symptomatic adult with an uninfected spouse and uninfected children would be much more likely to do so. Here, we have not incorporated this level of detail: we have shown that moderate average compliance could bring about substantial population-level benefits. Also, the nature of compliance behavior may change over the course of the outbreak. With these issues in mind, the quarantine and isolation process investigated here is being refined as a topic of ongoing investigation. In parallel, there is a clear need for psycho-behavioral surveillance studies [22] to estimate likely levels of compliance and to identify factors that would influence the decisions of individuals and households.

Implicit in the results presented here is the assumption that reducing the first-wave attack rate should be the primary goal of influenza preparedness planning. When complete transmission control is not achieved, this necessarily implies a longer epidemic. If the mortality rate of the pandemic strain is considered to be low in the local context, it is likely that some governments will place greater priority on reducing the duration of the outbreak than on reducing the number of infections. We suggest that designing policy for a longer period of societal disruption may be justified not only by reduced mortality but also by reduced peak stresses on the society as a whole. For example, for the baseline case, we found that QIA could reduce the peak incidence of infection from 3.7% to 0.8%. Although such analyses are beyond the scope of this work, the likelihood of maintaining uninterrupted key societal services (such as law enforcement, food distribution, and utility provision) may improve substantially across this range of reduction in infection incidence. Therefore, the potential massive adverse economic implications of a temporary breakdown may justify extending the expected period of disruption.

We have focused on measures to increase social distance that will consume substantial resources and for which detailed planning is required. To allow quarantined individuals to remain at home, provision must be made for food, water, and medicines to be delivered. This may be achieved through a central system or a neighborhood assistance scheme. For isolation, careful planning and investment would be required so that large facilities can be made operational in time to reduce transmission in the early stages of the epidemic. For anti-virals to be provided efficiently, a dedicated distribution system may be required. A recent review [23] suggests that household quarantine was not successfully implemented on any significant scale during the 1918 city-level epidemics upon which estimates of transmissibility are based [2,4]. Therefore, we suggest that the likely impact of the interventions we describe here is real and not already incorporated into estimates of transmissibility. Further, we suggest that modern transport and communication infrastructures are sufficiently more advanced than those available in 1918 that it is reasonable to expect that such interventions can now succeed.

Many countries have put in place formal pandemic preparedness plans following a framework set out by the World Health Organization. These national plans do mention the interventions included here, but they do not yet specify how these intervention processes will be implemented in even the broadest terms, nor do they attempt to predict the levels of resources required. We believe that our findings, and future studies that match detailed descriptions of interventions with realistic transmission models, can help to inform pandemic preparedness plans by quantifying both the benefits of, and resources required for, household-based interventions against pandemic influenza.

## Supporting Information

Figure S1 Time-Dependent Infection Import Rate (26 KB PDF)

Figure S2 Household Dynamics (20 KB PDF)

Figure S3 The Basic Reproductive Number (37 KB PDF)

Figure S4 Numbers of Episodes of Quarantine by Household (21 KB PDF)

Figure S5 Multiple Scenarios for Testing Sensitivity and Capacity (24 KB PDF)

Figure S6 Impact of a Delay in the Start of Interventions (29 KB PDF)

Protocol S1 Model Definition (83 KB PDF)

Table S1 Impact of Voluntary Household Quarantine on Households of Different Sizes (21 KB PDF)

## Abbreviations

IAR: infection attack rate
Q: voluntary household quarantine
QA: voluntary household quarantine and anti-virals
QI: voluntary household quarantine and isolation
QIA: voluntary household quarantine, isolation, and anti-virals
QIAC: voluntary household quarantine, isolation, anti-virals, and contact tracing
