# Supplementary material for: Reducing the Impact of the Next Influenza Pandemic Using Household-Based Public Health Interventions
Source: PLoS Med. 2006 Aug 8;3(9):e361. doi: 10.1371/journal.pmed.0030361 (PMC1526768; doi:10.1371/journal.pmed.0030361)
Supplement: Supplementary file 1 [file pmed.0030361.sd001.pdf]

Supporting Protocol S1: Model definition

Disease transmission

The transmission model was individual-based and simulations were conducted in continuous time. The population was made up of N people all of whom were in one of the disease states defined in Figure 1. The model progressed by simulating the time of the next infection and choosing the individual to which that infection occurred. After infection, disease progression within an individual was determined by transition events such as “become infectious” and “become symptomatic”, as per the waiting time distributions defined in Figure 1 and Table 1. New infection events were generated by evaluating the instantaneous force of infection for each individual, λ_i, drawing all waiting times from the appropriate exponential distributions and so simulating the time and individual associated with the next infection event. Intervention events were determined by the algorithm defined in the Methods section of the main text (or defined below when testing was in effect). If a disease progression event or an intervention event occurred before the next infection, these events took precedence and the next infection event was recalculated starting from that point in time. Otherwise, time was updated to the next infection event, the identified individual was infected and the process of generating the next event was started again. Key simulation results were confirmed using independent source code and the dynamics of the mass-action sub-model were verified using a deterministic differential equation model.

Individual i, $1 \le i \le N$, was a member of a household of size n_i^H and of a peer-group of size n_i^{PG}. Each household size n^H was drawn from distribution H and peer-group n^{PG} from distribution P. Note that the average value of n_i^H over all individuals was different from the average value of n^H over all households as more people live in larger households. The number of active household members in the household of the ith individual, i.e. not isolated or dead, was defined to be n_i^{HA}. The underlying coefficient of transmission was defined to be β with relative hazards h_{AS} (asymptomatic to symptomatic), h_{PS} (presymptomatic to symptomatic), h_{PH} (peer-

group to household), and h_{CH} (community to household), used as modifiers for different disease stages and transmission settings. Values for β and the relative hazards were derived from other more intuitive parameters (see below in this section). A further modifier ε was defined to be the probability that people stayed away from work or school and do not mix with their peer-groups when symptomatic. Note that they did mix with the community as a whole when symptomatic. This tendency to stay at home was considered to be part of the natural behavioral response to influenza infection and not motivated by any particular public health directive.

Two indicator functions are required to allow a clear definition of the force of infection. Let δ_{ij}^{H} be equal to 1 if two distinct individuals i and j share a household and equal to 0 otherwise. Similarly, let δ_{ij}^{PG} be the indicator function for peer-groups. The instantaneous force of infection experienced by the ith individual if they were not in isolation was

$$\lambda_i = \sum_{j=1}^{j=N} \left\{ \delta_{ij}^{H} \frac{m_j \beta}{n_i^{HA}} + \delta_{ij}^{PG} m_j \varepsilon_j h_{PH} \beta + \frac{m_j h_{CH} \beta}{N} \right\},$$

where

$$m_j = \begin{cases} 1 \text{ if } j \text{ was symptomatic,} \\ h_{PS} \text{ if } j \text{ was presymptomatic,} \\ h_{AS} \text{ if } j \text{ was asymptomatic,} \\ 0 \text{ otherwise,} \end{cases}$$

and

$$\varepsilon_j = \begin{cases} 0 \text{ with probability } \varepsilon_A \text{ if } j \text{ was an adult and was symptomatic} \\ 0 \text{ with probability } \varepsilon_C \text{ if } j \text{ was a child and was symptomatic} \\ 1 \text{ otherwise.} \end{cases}$$

When individuals left the home to enter isolation they no longer contributed to infection in the home, the peer-group or in the community. As a neutral assumption, transmission did still occur between those in isolation, but it was sufficiently well controlled so as not to be a significant factor for the whole population. The instantaneous force of infection experienced by ith individual if they were in isolation was

$$\lambda_i = \frac{\varepsilon_I R_0^*}{(E[D_S] + p_H E[D_H])} \frac{I_{\text{Isol}}}{N_{\text{Isol}}}$$

where I_{Isol} was the number of infectious individuals in isolation, N_{Isol} was the total number in isolation, ε_I was the ratio of transmissibility in isolation compared with the overall level of transmission outside isolation, D_S was the duration of symptoms, p_H was the probability of hospitalization conditional on being symptomatic and D_H was the duration of hospital stay.

Natural history parameters

Although hazards can be useful in some contexts for describing the natural history of infectious disease, we choose to present transmission scenarios in terms of the transmissibility. For example, we use θ, the proportion of transmission which is either presymptomatic or asymptomatic[1], rather than the relative hazards h_{PS} and h_{AS}. In this section, we define these parameters in terms of the average number of secondary cases (see Supporting Figure S3).

Let $\phi_P(h)$ be the probability that an infection event does not occur between two individuals in the simulation during the presymptomatic phase given that there was a constant hazard of infection h. Similarly, let $\phi_S(h)$ and $\phi_A(h)$ be the probabilities of no infection during the symptomatic and asymptomatic phases respectively. The probability of an infection being asymptomatic was defined to be p_A, the proportion of the population that were adults was q_A and the probability that a member of the population lived in a household of size n was equal to p_n. The average number of coworkers and classmates are denoted by n_w and n_c respectively. The average number of secondary infections generated by a single infectious individual, chosen at random, for disease stage X and transmission setting Y is r_{XY} where X is one of {P - presymptomatic, S - symptomatic or A - asymptomatic} and Y is one of {H - household, P - peer-group or C - community}. With reference to the definition of the force of infection above, individual expected numbers of secondary cases for different combinations of disease stage and setting are defined below. We assumed a maximum household size of 10

$$
\begin{aligned}
r_{PH} &= \sum_{n=1}^{10} p_n (n-1)\left[1-\phi_P\left(\frac{h_{PS}\beta}{n}\right)\right],\\
r_{AH} &= p_A \sum_{n=1}^{10} p_n (n-1)\phi_P\left(\frac{h_{PS}\beta}{n}\right)\left[1-\phi_A\left(\frac{h_{AS}\beta}{n}\right)\right],\\
r_{SH} &= (1-p_A)\sum_{n=1}^{10} p_n (n-1)\phi_P\left(\frac{h_{PS}\beta}{n}\right)\left[1-\phi_S\left(\frac{\beta}{n}\right)\right],\\
r_{PP} &= \left[(1-q_A)n_C + q_A n_W\right]\left[1-\phi_P\left(h_{PS}h_{PH}\beta\right)\right],\\
r_{AP} &= p_A\left[(1-q_A)n_C + q_A n_W\right]\phi_P\left(h_{PS}h_{PH}\beta\right)\left[1-\phi_A\left(h_{AS}h_{PH}\beta\right)\right],\\
r_{SP} &= (1-p_A)\left[(1-q_A)n_C + q_A n_W\right]\phi_P\left(h_{PS}h_{PH}\beta\right)\left[1-\phi_S\left(h_{PH}\beta\right)\right],\\
r_{PC} &= N\left[1-\phi_P\left(\frac{h_{PS}h_{CH}\beta}{N}\right)\right],\\
r_{AC} &= p_A N\phi_P\left(\frac{h_{PS}h_{CH}\beta}{N}\right)\left[1-\phi_A\left(\frac{h_{AS}h_{CH}\beta}{N}\right)\right],\\
r_{SC} &= (1-p_A)N\phi_P\left(\frac{h_{PS}h_{CH}\beta}{N}\right)\left[1-\phi_S\left(\frac{h_{CH}\beta}{N}\right)\right].
\end{aligned}
$$

The statistics introduced in Table 1 are defined in terms of generation time and the following quantities:

R_0^*	=	$\sum_{Y\in\{H,P,C\}}\sum_{X\in\{P,A,S\}} r_{XY}$	Average number of secondary cases generated by an infectious individual chosen at random in an otherwise susceptible population
θ	=	$\dfrac{\sum_{Y\in\{H,P,C\}}\sum_{X\in\{P,A\}} r_{XY}}{R_0}$	Proportion of transmission which is either presymptomatic or asymptomatic
ω	=	$\dfrac{\sum_{Y\in\{H,P,C\}} r_{AY} + p_A \sum_{Y\in\{H,P,C\}} r_{PY}}{R_0}$	Proportion of infections generated by those who are never symptomatic
γ	=	$\dfrac{\sum_{Y\in\{P,C\}}\sum_{X\in\{P,A,S\}} r_{XY}}{R_0}$	Proportion of transmission which occurs outside the home
δ	=	$\dfrac{\sum_{X\in\{P,A,S\}} r_{XC}}{\sum_{Y\in\{P,C\}}\sum_{X\in\{P,A,S\}} r_{XY}}$	Proportion of transmission outside the home that occurs in the community rather than the peer-group

Standard non-linear numerical techniques were used to obtain values for the relative hazards from these transmission parameters. For the base case (Table 1), the values of the hazards were as follows: the baseline hazard of infection between household members (multiplied by the household size) β = 0.525; the hazard during the presymptomatic phase relative to that during the symptomatic phase h_{PS} = 0.6544; the hazard for asymptomatic individuals relative to symptomatic individuals h_{AS} = 0.2237; the hazard for the peer-group relative to the home h_{PH} = 0.037; and the hazard of the community relative to the home (normalized for different population sizes) h_{CH} = 0.58. Note that as we assume 50% of adults and 100% of children do not mix in their peer-group when symptomatic (see Table 1).

Generation time

Let T_i^{XY} be the time it takes the index case to infect his i th infectee in mixing group $Y \in \{H, P, C\}$ during disease stage $X \in \{P, A, S\}$. Similarly, let M_{XY} be the number of individuals in mixing group $Y \in \{H, P, C\}$ that the index case infects during disease stage $X \in \{P, A, S\}$. Define the generation time as

$$T_g = \frac{E\left[\sum_{Y \in \{H,P,C\}} \sum_{X \in \{P,A,S\}} \sum_{i=1}^{M_{XY}} T_i^{XY}\right]}{R_0}$$

For a given disease stage duration D_X with pdf f_X, where $X \in \{P, A, S\}$, define the following notation:

$$\phi_X(h) = E\left[e^{-hD_X}\right] = \int_0^\infty e^{-ht} f_X(t)dt$$

$$\alpha_X(h) = E\left[D_X e^{-hD_X}\right] = \int_0^\infty t e^{-ht} f_X(t)dt$$

Let B indicates the event that the index case will be symptomatic and N_H be the household size of the index case. For conciseness, we show the formulas for $E\left[\sum_{i=1}^{M_{PH}} T_i^{PH}\right]$ and $E\left[\sum_{i=1}^{M_{SH}} T_i^{SH} \mid B\right]$ only:

$$E\left[\sum_{i=1}^{M_{PH}} T_i^{PH}\right] = E\left[E\left[\sum_{i=1}^{M_{PH}} T_i^{PH} \mid N_H\right]\right]$$

$$= E\left[N_H\left(E\left[D_E\right]\left(1-\phi_P\left(\frac{h_{PS}\beta}{N_H}\right)\right)+\frac{1}{2}\left(E\left[D_P\right]-\alpha_P\left(\frac{h_{PS}\beta}{N_H}\right)\right)\right)\right]$$

$$E\left[\sum_{i=1}^{M_{SH}} T_i^{SH} \mid B\right] = E\left[E\left[\sum_{i=1}^{M_{SH}} T_i^{SH} \mid N_H, B\right] \mid B\right]$$

$$= E\left[N_H \left(\begin{array}{l}\left(E[D_E]\phi_P\left(\frac{h_{PS}\beta}{N_H}\right) + \alpha_P\left(\frac{h_{PS}\beta}{N_H}\right)\right)\left(1-\phi_S\left(\frac{\beta}{N_H}\right)\right) + \\ \frac{1}{2}\phi_P\left(\frac{h_{PS}\beta}{N_H}\right)\left(E[D_S] - \alpha_S\left(\frac{\beta}{N_H}\right)\right)\end{array}\right) \mid B\right]$$

Testing

The maximum number of tests that could be processed in single day was 3,000 per 6.8 million population in the "constrained" case in Supporting Figure S5. The time it takes to test a sample was assumed to be 1.5 days and the sensitivity and specificity of the test was assumed to be 60% and 100% respectively. These approximate values are based on maximum use of current laboratory infrastructure in Hong Kong. Although laboratory sensitivity would be much higher than 60%, it is likely that many samples taken in the field would not be viable.

The voluntary household intervention process was changed to the following when testing was in effect:

1. If testing was in effect, testing was closed to new jobs when there were fewer than 10 available test servers. Testing remained closed for 1 day or until more than 10 free test servers were available, whichever occurred last.
2. An individual from households not in voluntary quarantine had the opportunity to enter the program via one of the following three routes: she developed symptoms, she was contacted through contact tracing or she was hospitalized. We assumed she volunteered and actually reported with probability p_C for symptoms and contact tracing, and with probability 1 for hospitalization. She complied with the program until released. After release, individuals were not bound by previous decisions to join or not join, i.e. they could choose again.
3. Each other member of her household complied with intervention instructions with probability p_C. If testing was open, a sample was taken from each compliant household member. Test results would return in 1.5 days.

4. After a delay of 1 day, all compliant non-symptomatic household members took 1 dose of prophylactic antivirals per day when antiviral policies were in effect (QA,QIA,QIAC). Symptomatic household members took 2 doses of anitvirals per day.
5. If contact tracing was in effect, each compliant adult member of the household named, on average, 5 people from her peer-group if she had not been asked to name contacts before.
6. If isolation was in effect, newly found symptomatic individuals who were compliant voluntarily entered isolation with probability p_C after a delay of 1 day. If an isolated individual no longer showed symptoms (or no longer had detectable viral load if testing was in effect) after 3 days, she was released from isolation and joined her household, which might be quarantined. Otherwise, she was isolated for another 3 days.
7. Isolated individuals were given 2 doses of antivirals per day, without a delay, in all simulations, regardless of the policy for the use of antivirals in households.
8. If contact tracing was in effect, contacts (if known and not already in the program), of all newly found symptomatic or hospitalized household members were traced with a mean delay of 1 day.
9. If the household was tested, the household was released from quarantine if all test results were negative and there were no new symptoms or hospitalizations since the samples were taken. If the household was not releasable, they were retained for 4 days before retesting. If there were new symptoms while waiting for retesting, retesting was voided and the household was quarantined for 7 days and then Step 8 was taken. If testing was closed at the time of retesting, the household was considered for release using the symptoms-based rule in Step 8.
10. If testing was not in effect or closed, and if there were no new symptoms (from compliant members) or hospitalizations in the household for 7 (or 4 days if this was reached from Step 7 when testing was closed to retesting) days, the household was released from quarantine. Otherwise, we returned to Step 6 at the time new symptoms or hospitalizations occurred.

The peer-group contact network

We assume that both children and adults are members of a highly connected groups of peers. For children, this corresponds directly with their classmates at school. For adults this group represents their co-workers or day-time social contacts. We constrained our contact tracing process to ask for only 5 contacts from each compliant symptomatic adult in the program and we assumed that the effective average size of the peer group was 30. These assumptions are conservative as they resulted in a low average probability that a named contact was actually infected by the individual. However, the use of non-overlapping peer-groups for adults may overestimate levels of connectivity and hence may overestimate the efficacy of contact tracing.

Reference

1. Fraser C, Riley S, Anderson RM, Ferguson NM (2004) Factors that make an infectious disease outbreak controllable. Proceedings Of The National Academy Of Sciences Of The United States Of America 101: 6146-6151.
