# Supplementary material for: Reducing the Impact of the Next Influenza Pandemic Using Household-Based Public Health Interventions
Source: PLoS Med. 2006 Aug 8;3(9):e361. doi: 10.1371/journal.pmed.0030361 (PMC1526768; doi:10.1371/journal.pmed.0030361)
Supplement: Supplementary file 2 [file pmed.0030361.sg001.pdf]

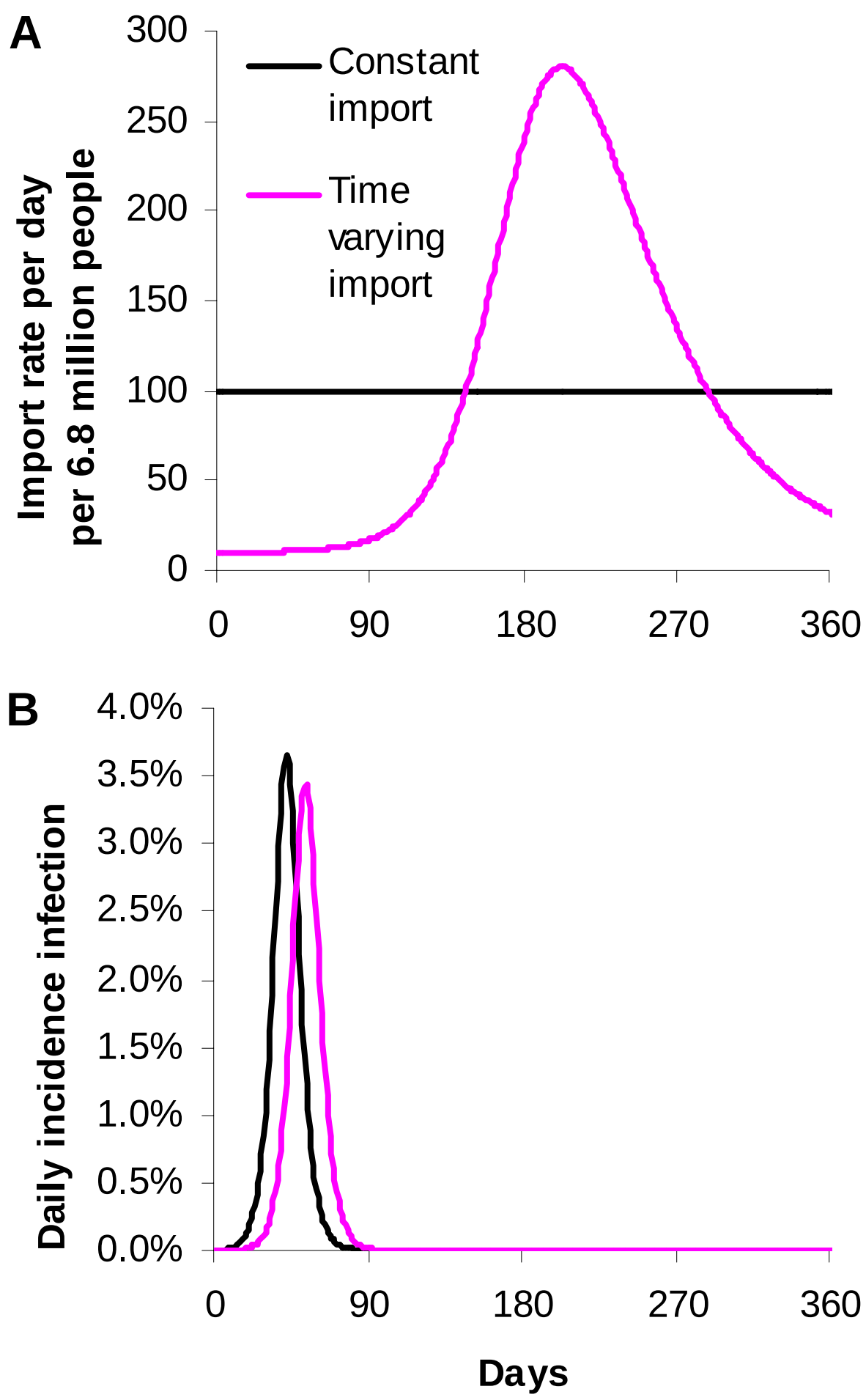

Supporting Figure S1. Time dependent import rate. **A** compares the constant import rate of 1.5 infectious people per 100,00 per day used for other results against a time varying import rate. For the time varying import rate, the parameters of the classic (SIR) epidemic model[1] are tuned such that its peak incidence of 4 infectious people per 100,000 per day occurs at around the 200th day. The amplitude of the epidemic import rate is set such that the total number of imports over 365 days is the same as in the case of a constant import rate. **B** shows the predicted disease incidence. Progression of the outbreak is similar in both cases once there are enough infectious individuals in the population. The shape of the import rate function has little impact on outbreak progression once widespread local transmission occurs.

Reference

1. Anderson RM, May RM (1991) Infectious diseases of humans. Oxford: Oxford University Press.
