# Supplementary material for: Reducing the Impact of the Next Influenza Pandemic Using Household-Based Public Health Interventions
Source: PLoS Med. 2006 Aug 8;3(9):e361. doi: 10.1371/journal.pmed.0030361 (PMC1526768; doi:10.1371/journal.pmed.0030361)
Supplement: Supplementary file 3 [file pmed.0030361.sg002.pdf]

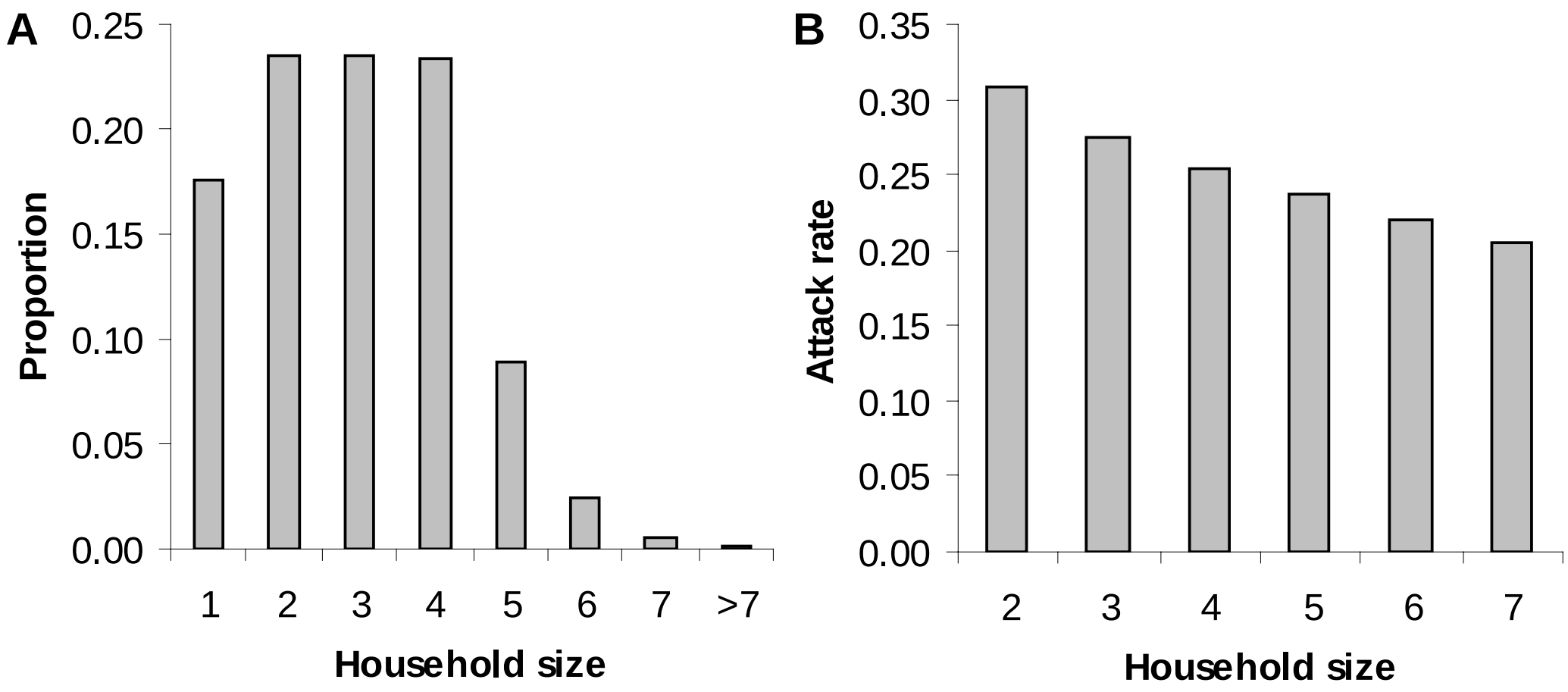

Supporting Figure S2. Household dynamics. **A** shows the distribution of household sizes in Hong Kong[1]. **B** shows household infection attack rate for different household sizes averaged over 500 model realizations for each household size for the baseline transmission scenario (see main text) without transmission between households. The overall household attack rate of 22.5% in the absence of community transmission implies a symptomatic attack rate of 15.2%.

Reference

1. Leung GM, Wong IO, Chan WS, Choi S, Lo SV (2005) The ecology of health care in Hong Kong. Soc Sci Med 61: 577-590.
