# Supplementary material for: Reducing the Impact of the Next Influenza Pandemic Using Household-Based Public Health Interventions
Source: PLoS Med. 2006 Aug 8;3(9):e361. doi: 10.1371/journal.pmed.0030361 (PMC1526768; doi:10.1371/journal.pmed.0030361)
Supplement: Supplementary file 4 [file pmed.0030361.sg003.pdf]

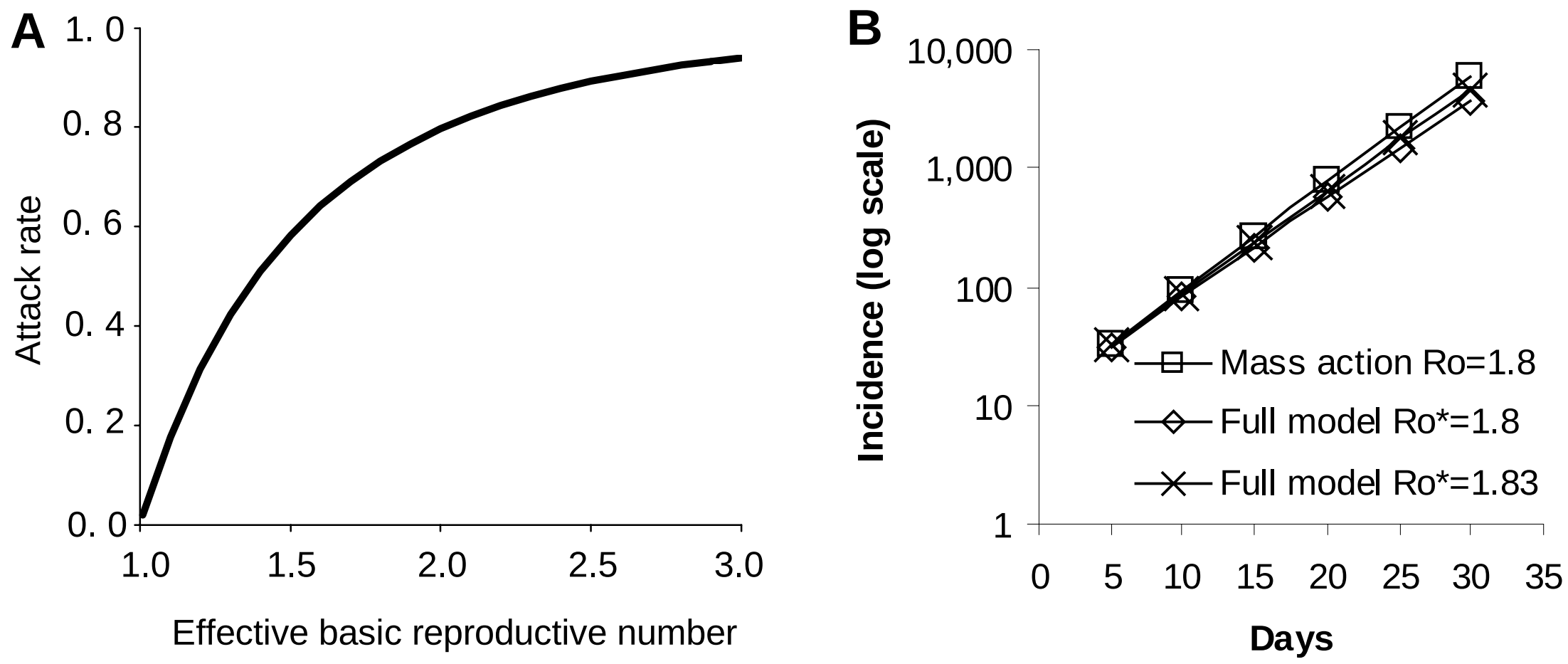

Supporting Figure S3. The basic reproductive number. **A** the relationship between the effective basic reproductive number, R_0^E, and the infection attack rate (IAR). Making very few assumptions about the natural history of the disease[1,2], the IAR when $R_0^E > 1$ is the root (falling between 0 and 1) of the following characteristic equation $\ln(1-\text{IAR}) = -R_0^E\text{IAR}$. **B** Calibration of the basic reproductive number used in the model. The basic reproductive number R_0 is defined to be the average number of infections generated by a *typically* infectious individual in an otherwise susceptible population[3]. We define the "average number of secondary cases" R_0^* to be the average number of secondary cases generated by an individual *chosen at random* in an otherwise susceptible population. The difference between these two definitions is somewhat technical, but can make a material difference in practice. It hinges on the word "typically" which can be taken to mean[1] "chosen with probability proportional to the components of the eigenvector associated with the largest eigenvalue of the next generation operator". If transmission is only allowed along the community route (i.e. mass-action, no household or peer-group transmission) the average of our model attack rate converges with those of the classic epidemic size calculation, i.e. $R_0 = R_0^*$ as would be expected. Generally, if one accepts that R_0 is defined for such models[4], additional population structure such as households and peer-groups introduces a discrepancy between R_0 and R_0^*, with $R_0 > R_0^*$. In this figure, we show that for the full model at baseline transmission (including household, peer-group and community transmission), an R_0^*=1.8 underestimates the mass-action R_0 of 1.8. An R_0^* = 1.83 is empirically equivalent to an R_0 of 1.8 in terms of final attack rate. Almost all of the discrepancy is generated by household structure. Therefore, for sensitivity analysis (Figure 3) we approximate R_0^* with 1.83/1.8* R_0. Note that R_0^* has a threshold value at 1.045 (not 1.83/1.8 = 1.017) as the relationship between R_0 and R_0^* is not linear.

References

1. Diekmann O, Heesterbeeck JAP (2002) Mathematical Epidemiology of Infectious Diseases: Model Building, Analysis and Interpretation. New York: Wiley.
2. Kermack WO, McKendrick AG (1927) A Contribution to the Mathematical Theory of Epidemics. Proceedings Of The Royal Society Of London Series A Containing papers of a Mathematical and Physical Character 115: 700-721.
3. Heesterbeek JAP (2002) A brief history of R-0 and a recipe for its calculation. Acta Biotheoretica 50: 189-204.
4. Ball FG, Britton T, Lyne OD (2004) Stochastic multitype epidemics in a community of households: Estimation of threshold parameter R-* and secure vaccination coverage. Biometrika 91: 345-362.
