# Supplementary material for: Reducing the Impact of the Next Influenza Pandemic Using Household-Based Public Health Interventions
Source: PLoS Med. 2006 Aug 8;3(9):e361. doi: 10.1371/journal.pmed.0030361 (PMC1526768; doi:10.1371/journal.pmed.0030361)
Supplement: Supplementary file 5 [file pmed.0030361.sg004.pdf]

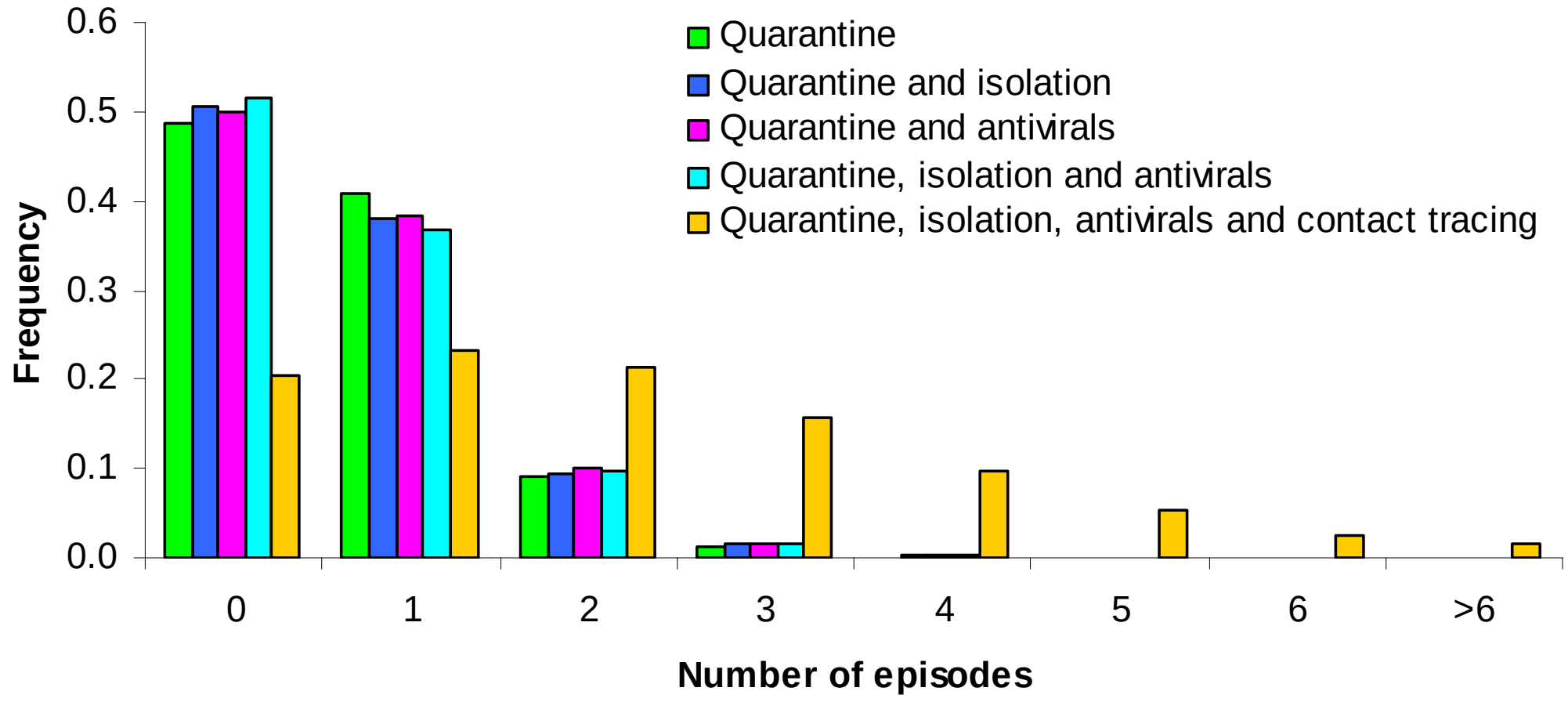

Supporting Figure S4. Numbers of episodes of quarantine by household. The chart shows the distribution of numbers of episodes of quarantine for different intervention policies. Results are averages of 100 model realizations in a population of 1 million individuals. The average duration of quarantine ranged between 7.2 and 8.2 days per episode for the five policies. There was little variation in this average for households which underwent different numbers of episodes. The addition of contact tracing substantially increases the average number of quarantine episodes experienced by each household: the average for non-contact tracing policies was 0.6 episodes. When quarantine, isolation, antivirals and contact tracing were employed, the average rose to 2.0 episodes.
