# Supplementary material for: Reducing the Impact of the Next Influenza Pandemic Using Household-Based Public Health Interventions
Source: PLoS Med. 2006 Aug 8;3(9):e361. doi: 10.1371/journal.pmed.0030361 (PMC1526768; doi:10.1371/journal.pmed.0030361)
Supplement: Supplementary file 6 [file pmed.0030361.sg005.pdf]

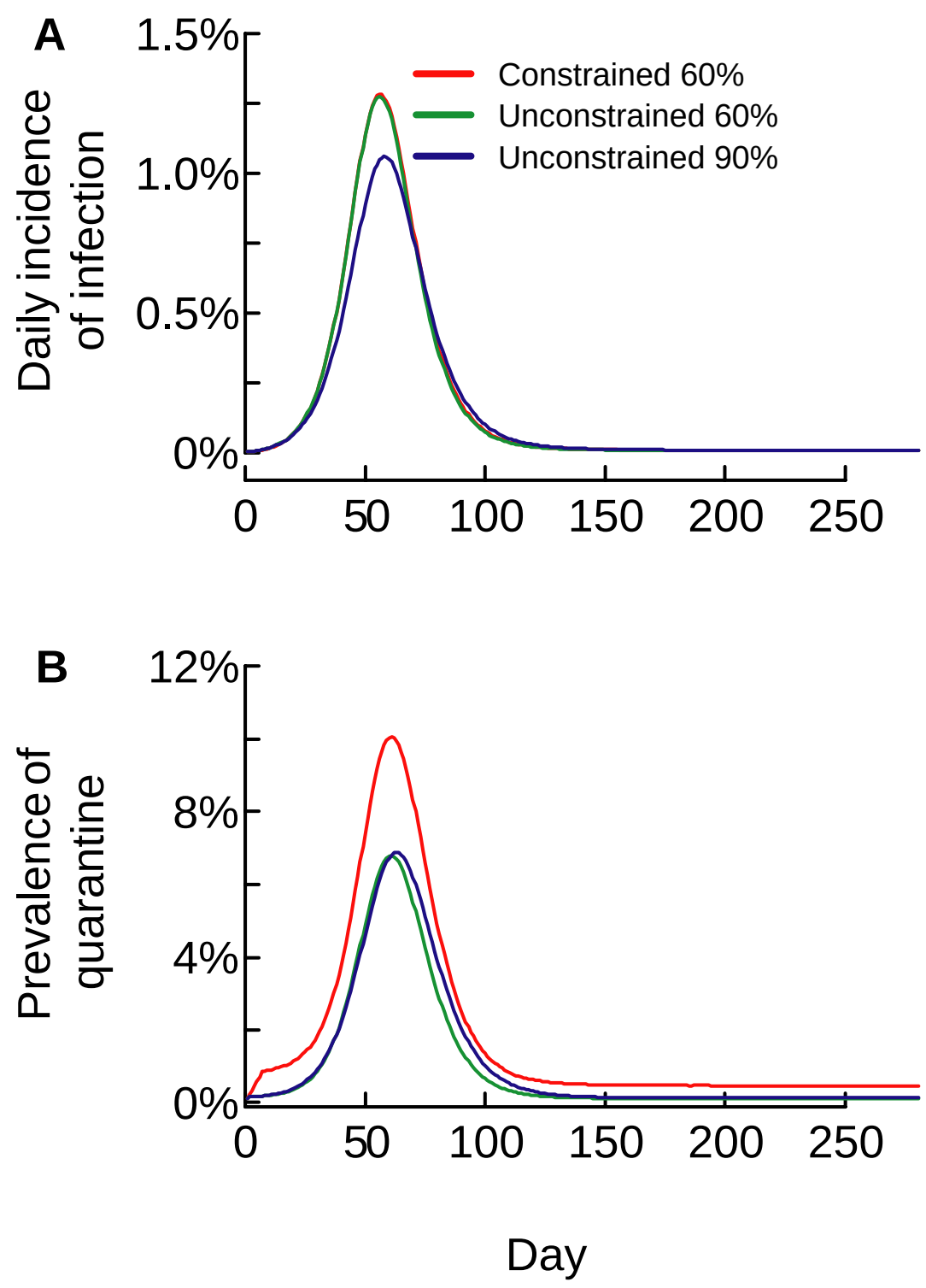


**Supporting Figure S5.** Multiple scenarios for testing sensitivity and capacity. Our baseline assumptions for testing was constrained to at most 3,000 per 6.8 million people per 1.5 days at 60% sensitivity. The two other scenarios investigated here are: unconstrained capacity with 60% sensitivity and unconstrained capacity with 90% sensitivity. **A** shows the infection incidence. **B** shows the percentage of the population living in homes that were quarantined. All results are averages of 100 model realizations in a population of 1 million individuals.
