# Supplementary material for: Reducing the Impact of the Next Influenza Pandemic Using Household-Based Public Health Interventions
Source: PLoS Med. 2006 Aug 8;3(9):e361. doi: 10.1371/journal.pmed.0030361 (PMC1526768; doi:10.1371/journal.pmed.0030361)
Supplement: Supplementary file 7 [file pmed.0030361.sg006.pdf]

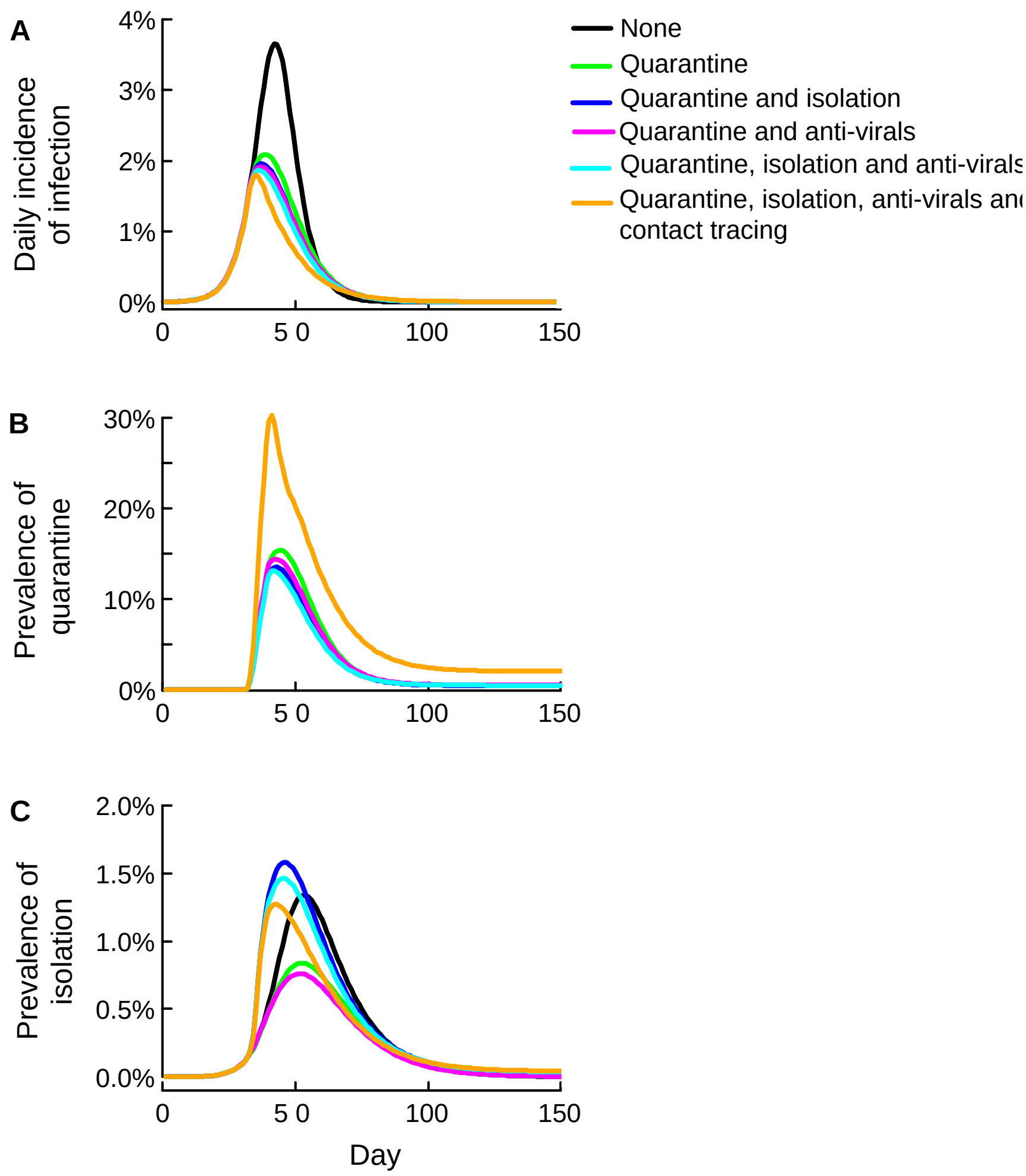

Supporting Figure S6. Impact of a delay in the start of interventions. The charts above show the sensitivity of our results to the assumption that all interventions are in place prior to the first importation of the pandemic strain of influenza. The scenarios are the same as those presented in Figure 2 except that the start of all interventions was delayed until 5% of the population have experienced symptoms of pandemic influenza (7.5% infected). Baseline transmission (None) and five intervention scenarios: voluntary household quarantine (Q); voluntary household quarantine and isolation (QI); voluntary household quarantine and anti-virals (QA); voluntary household quarantine, isolation and anti-virals (QIA); and voluntary household quarantine, isolation, anti-virals and contact tracing (QIAC). **A** shows the incidence of infection, **B** the percentage of the population living in homes that were quarantined and **C** the percentage of the population in isolation.Note that the efficacy of interventions, once initiated, was not substantially affected by the delayed start. For example, the overall attack rate for quarantine alone (Q) with the delayed start was 54%. This corresponds to an attack rate of 50% of the population not yet infected when the interventions started. This compares favorably with the overall attack rate for Q of 49% when the start of interventions is not delayed (see main text).
