# Supplementary material for: Reducing the Impact of the Next Influenza Pandemic Using Household-Based Public Health Interventions
Source: PLoS Med. 2006 Aug 8;3(9):e361. doi: 10.1371/journal.pmed.0030361 (PMC1526768; doi:10.1371/journal.pmed.0030361)
Supplement: Supplementary file 8 [file pmed.0030361.st001.pdf]

Supporting Table S1. Legend on next page.

Size of infector's household	Symptomatic status of household index	Setting of infection event	Average number of infections per 100,000 with no intervention (None)	Average number of infections per 100,000 with voluntary quarantine (Q, 50% compliance)	Q as a percentage of None
1	Asymptomatic	Household	0	0	-
		Peer-group	261	187	71%
		Community	144	101	70%
	Symptomatic	Household	0	0	-
		Peer-group	1,135	600	53%
		Community	1,153	477	41%
2	Asymptomatic	Household	340	238	70%
		Peer-group	928	603	65%
		Community	654	360	55%
	Symptomatic	Household	1,897	1,531	81%
		Peer-group	3,336	1,945	58%
		Community	3,323	1,520	46%
3	Asymptomatic	Household	963	675	70%
		Peer-group	1,538	990	64%
		Community	1,213	623	51%
	Symptomatic	Household	4,010	3,577	89%
		Peer-group	4,715	2,925	62%
		Community	4,974	2,399	48%
4	Asymptomatic	Household	1,763	1,299	74%
		Peer-group	2,161	1,402	65%
		Community	1,836	927	51%
	Symptomatic	Household	6,179	5,923	96%
		Peer-group	6,034	3,896	65%
		Community	6,606	3,305	50%
5	Asymptomatic	Household	1,029	810	79%
		Peer-group	1,102	716	65%
		Community	973	490	50%
	Symptomatic	Household	3,215	3,252	101%
		Peer-group	2,953	1,951	66%
		Community	3,184	1,632	51%
6	Asymptomatic	Household	396	330	83%
		Peer-group	385	253	66%
		Community	353	178	51%
	Symptomatic	Household	1,148	1,209	105%
		Peer-group	996	670	67%
		Community	1,077	563	52%
7 or more	Asymptomatic	Household	151	137	90%
		Peer-group	135	90	67%
		Community	127	66	52%
	Symptomatic	Household	410	450	110%
		Peer-group	337	231	68%
		Community	367	194	53%
**Total**			**73,499**	**48,723**	**66%**
**Attack rate**			**73%**	**49%**	

Supporting Table S1 (Table on previous page). Household quarantine with 50% compliance worked because it was very effective for some “high volume” households. The effect depends on household size, on the symptomatic status of the household index and on the source of infection. Infections are classified by the household size of the infector and by the symptomatic status of the index case of the household of the infector. All values are expected numbers of infections per 100,000 people and are averages of 100 model simulations in a population of 1 million with the baseline transmission parameter values (see Table 1). Infections are further segregated by type: household, peer-group and community. For example, without interventions, the expected number of infections into the community from households of size 4, where the index case was symptomatic, was 6,606 per 100,000. This value is driven by the large number of households of size 4 and by the relative infectivity of those households. When voluntary household quarantine was in effect, the number of infections per 100,000 dropped to 3,305. The drop in the number of infections to only 50% of the non-intervention value (for household size 4, index case symptomatic, and community source) shows how the most simple policy (voluntary household quarantine) would still be effective even with only moderate individual-level compliance.
